# Viewing the Kenyan health system through an equity lens: implications for universal coverage

**DOI:** 10.1186/1475-9276-10-22

**Published:** 2011-05-26

**Authors:** Jane Chuma, Vincent Okungu

**Affiliations:** 1 Kenya Medical Research Institute-Wellcome Trust Research Programme, P.O Box 230, Kilifi, Kenya; 2 Centre for Tropical Medicine, Nuffield Department of Clinical Medicine, University of Oxford, UK

## Abstract

**Introduction:**

Equity and universal coverage currently dominate policy debates worldwide. Health financing approaches are central to universal coverage. The way funds are collected, pooled, and used to purchase or provide services should be carefully considered to ensure that population needs are addressed under a universal health system. The aim of this paper is to assess the extent to which the Kenyan health financing system meets the key requirements for universal coverage, including income and risk cross-subsidisation. Recommendations on how to address existing equity challenges and progress towards universal coverage are made.

**Methods:**

An extensive review of published and gray literature was conducted to identify the sources of health care funds in Kenya. Documents were mainly sourced from the Ministry of Medical Services and the Ministry of Public Health and Sanitation. Country level documents were the main sources of data. In cases where data were not available at the country level, they were sought from the World Health Organisation website. Each financing mechanism was analysed in respect to key functions namely, revenue generation, pooling and purchasing.

**Results:**

The Kenyan health sector relies heavily on out-of-pocket payments. Government funds are mainly allocated through historical incremental approach. The sector is largely underfunded and health care contributions are regressive (i.e. the poor contribute a larger proportion of their income to health care than the rich). Health financing in Kenya is fragmented and there is very limited risk and income cross-subsidisation. The country has made little progress towards achieving international benchmarks including the Abuja target of allocating 15% of government's budget to the health sector.

**Conclusions:**

The Kenyan health system is highly inequitable and policies aimed at promoting equity and addressing the needs of the poor and vulnerable have not been successful. Some progress has been made towards addressing equity challenges, but universal coverage will not be achieved unless the country adopts a systemic approach to health financing reforms. Such an approach should be informed by the wider health system goals of equity and efficiency.

## Introduction

Health care financing and equity currently dominate policy agendas worldwide [1,2]. Governments and international organisations are recognising that equitable health systems are essential to achieving health related millennium development goals, that financing approaches are critical for the performance of any health system and for achieving universal coverage [1-3]. Consequently, many low income countries, including Kenya, are considering how to reform their health financing systems in a way that promotes equity and efficiency.

In 2005, the 58^th ^World Health Assembly called for health systems to move towards universal coverage, where all individuals have access to "key promotive, preventive, curative and rehabilitative health interventions for all at an affordable cost, thereby achieving equity in access". It urged member states to ensure that health financing systems incorporate an element of pre-payment and risk pooling [1,4]. Universal health systems seek to be equitable in terms of delivery and financing. Equitable health financing requires that health care payments are on the basis of ability to pay; that there exists financial protection to ensure that everyone in need of health services is able to access them without putting people at risk of a financial catastrophe and that there are risk and income cross-subsidies (i.e. from the healthy to the ill and from the wealthy to the poor). Equitable delivery of health services ensures that people benefit from health services according to need for care [1,3]. Responding to the WHO call, the 56^th ^session of the regional committee for health in Africa urged member states to strengthen their national prepaid health financing systems, to develop comprehensive health financing policies and strategic plans and to build capacity for generating, disseminating and using evidence from health financing in decision making. They also called on the World Health Organization (WHO) to provide support to fair and sustainable financing and to identify financing approaches most suitable for the African region [5].

Health financing systems have three inter-related functions, which are central to achieving universal coverage (UC). They include revenue collection, pooling and purchasing [6]. Revenue collection refers to the process by which health systems receive money from households and organizations. Pooling refers to the accumulation and management of revenues to ensure that the risk of paying for health care is borne by all the members of the pool and not by each contributor individually. It embodies the insurance function within a health system. Pooling can be explicit or implicit: explicit, when people knowingly subscribe to a health insurance scheme; and implicit, where contributions are through tax revenue [6,7]. Purchasing is the process by which pooled funds are paid to providers in order to deliver a set of health interventions. It involves the transfer of pooled resources to service providers on behalf of the population for which the funds are pooled [6]. Purchasing can be strategic or passive [7]: strategic purchasing involves a continuous search for the best ways to maximise health systems performance by deciding which interventions should be purchased, while passive purchasing implies following a pre-determined budget or simply paying bills when presented. Strategic purchasing is best for universal coverage. In most cases, pooling and purchasing are implemented by the same organisation. Depending on how they are designed, payment mechanisms can influence provider behaviour [6]; they can act as incentive/disincentives to providers. Achieving UC will depend on the extent to which countries combine these functions to ensure there is equitable and efficient revenue generation, the extent to which financing systems encourage cross-subsidisation and the degree in which health systems provide or purchase effective health services for the population [1,7].

The aim of this paper is to assess the extent to which the Kenyan health financing system meets the key requirements for universal coverage including income and risk cross-subsidisation. Using the Kutzin framework [6], the paper demonstrates how the Kenyan health system performs the key financing functions and the implications of these arrangements for equity and UC. It also demonstrates the progress Kenya has made towards achieving internationally accepted benchmarks in health care financing and makes recommendations on how the country can progress towards universal coverage. The paper provides a comprehensive description of Kenya's health care financing system, how it has changed over time and the key equity concerns arising from current, past and upcoming health financing policies.

### History of health care financing in Kenya and implications for equity

Table 1 provides a summary of key health financing policy developments in Kenya. Following independence in 1963, the Kenyan post-colonial government made universal health care a major policy goal. Two years after independence, the post-colonial government abolished user fees that were implemented by the colonialist. Health services were funded primarily through general tax up to 1988, when the Kenyan government yielded to pressure from the World Bank and International Monetary Fund to introduce user fees and other major reforms in the health sector. Poor economic performance, inadequate financial resources and declining budgets were some of the reasons given to justify the introduction of user fees [8]. In the post colonial period, user fees were first introduced in 1989, but were suspended in 1990 and reintroduced in phases in 1991 [9]. Reasons for the failure of the 1989 implementation were attributed to various factors including: hurried implementation; massive declines in utilisation of health services; lack of quality improvements; and poor revenue collection [9-12]. Following the reintroduction in 1991, user fees were charged for individual services like drugs, injections, and laboratory services, instead of consultation as was previously the case. Revenue collected was returned to the district level to cater for public health needs within the district and facilities developed detailed plans for spending 75% of the revenue. A waiving policy to protect the poor was put in place, and children below five years were exempted from all charges, but in reality waiving mechanisms hardly existed [10].

**Table 1:** Development of health care financing policies in Kenya

Years	Policy	Equity impacts
Colonial period	User fees in all public facilities	Discriminative policy against Kenyans, imposed by colonial government

1963-1965	User fees initially introduced continued to exist for two years after independence	Negative impacts of affordability and utilisation of health care services

1965	User fees removed at all public health facilities. Health services provided for free and funded predominantly through tax revenue	Potential for equity provided there are mechanism to ensure that the poor benefit from tax funded system

1989	User fees introduced in all levels of care.	Negative impact on demand for health care especially among the poorest population; decreased utilisation including essential services like immunisation

1990	User fees suspended in all public health facilities. Waivers and exemption put in place to protect the poor and vulnerable. Failure linked to poor policy design and implementation.	Increase in utilisation patterns, confirming previous reports that user fees are a barrier to access.

1991-2003	User fees were re-introduced in 1991, through a phased implementation approach stating from hospital level. Children under five, special conditions/services like immunisation and tuberculosis were exempted from payment. User fees continued to exist in Kenya at all levels of care.	User fees major barrier to access, high out-of-pocket payment, catastrophic impacts, and negative implications for equity.

2004	User fees abolished at dispensaries and health centres (the lowest level of care), and instead a registration fees of Kenya shillings 10 and 20 respectively was introduced. Children under five, the poor, special conditions/services like malaria and tuberculosis were exempted from payment.	Utilisation increased by 70%; the large increased was not sustained, although in general utilisations was 30% higher than before user fee removal. Adherence to the policy has been low, due to cash shortages

2007	All fees for deliveries at public health facilities were abolished	No data on extent to which policy was implemented and no evaluation has taken place.

2010	A health sector services fund (HSSF) that compensates facilities for lost revenue associated with user fee removal introduced. Dispensaries and health centre receive funds directly into their bank accounts from the treasury.	Possible positive impacts on adherence to fee removal policy and equity.

User fees and other out-of-pocket payments (OOPs) have impacted negatively on utilisation of health care services in Kenya [12-15]. The majority of the population cannot afford to pay for health care, the poor are less likely to utilize health services when they are ill, and wide disparities in utilization exist between geographical regions and between urban and rural areas [14,15]. Socio-economic and geographic inequities are wider for inpatient care than outpatient care. Those who pay for care incur high costs that are sometimes catastrophic and adopt coping strategies with negative implications for their socio-economic status, while other simply fail to seek care [16,17].

In addition to user fees, the government encouraged development of the private health sector, a move that saw an upsurge in private health care providers in the country. Many private providers came up to respond to the demand for health care. Since public hospitals charged fees and were perceived to offer low quality care, people opted to pay for private services that were perceived to be of better quality. The private sector has since grown in Kenya, owning 49% of health services and regulating it remains a major challenge [18].

## Methods

The findings presented in this paper are from a review of both published and gray literature mainly sourced from the Ministry of Medical Services (MOMS) and Ministry of Public Health and Sanitation (MOPHS). Key documents reviewed included the Kenya national health accounts, Kenya health expenditure and utilisation survey reports and public expenditure reviews. Also reviewed to establish government commitment to equity were various policy documents including the Kenya health policy framework, the second national health sector strategic plan of 2005-2010, and the health policy and financing strategy. Country level estimates data were used except where the same were lacking at country level, in which case data were sought from international organisations websites including the WHO. Where large discrepancies existed between data reported at country level and those acquired from international organisations, we sought opinions from the ministries of health officials on the extent to which these data reflected reality.

Each financing mechanism was analysed in respect to key functions including revenue generation, pooling and purchasing [6]. All expenditure data were entered in Microsoft Excel spreadsheet, and bar charts and line graphs generated. Ethical approval was obtained from the Kenya Medical Research Institute (Protocol number 1609).

## Results

### Overview of health care financing and government health expenditure

Health care funds in Kenya come from the public (government), private (private companies and households) and donors. Out-of-pocket payments remain the largest source of health funds in Kenya, contributing 51.1% of total health expenditure (THE) in 2001/2002, and 35.9% in 2005/2006 [19,20]. Government spending on health accounted for 29.6% of THE in 2001/2002, and 29.3% in 2005/2006. Estimates of the 2008/2009 national health accounts indicate that this pattern might have changed, with the government contributing 35% of THE and households contributing 24.1%. Donors' contribution to the health sector in Kenya is relatively large and has increased dramatically in the last decade [19,20]. In 1994, donor funds only accounted for 8% of THE. This proportion increased to 16% in 2001/2002 and to 31.0% in 2005/2006. It is estimated that donor expenditure on health in 2008/09 amounted to 40.6% of THE [21]. Between 2001/2002 and 2005/2006, the total contributions of donor funds to total health expenditure increased from US$ 118.9 million to US$ 298.6 million. A large proportion of these funds (78%) went to funding HIV/AIDS related programmes[22].

## Revenue collection

### Source of funds and contribution methods

#### Government tax revenue and government spending on health

General tax revenue is generated from value added tax (30%); personal income tax (24%), company tax (14%), and fuel tax (13%). Import and excise duty each account for 10% of total revenue. Personal income tax is structured progressively, and therefore can be considered equitable. Contribution rates range from 10% for the lowest income bracket to 30% for the highest income brackets. Individuals earning less than Kenya shillings (KES) 133,620 per year are exempted from paying income tax. Value added tax (VAT) is charged at 16%. VAT contributions are likely to be regressive because prices of goods and services do not discriminate by income, although some goods are exempted from VAT payment. Corporate income tax ranges from 20% (for new companies) to 37.5% (for non-resident companies). A proportion of these taxes are allocated to funding health care services in the country.

**Figure 1:**
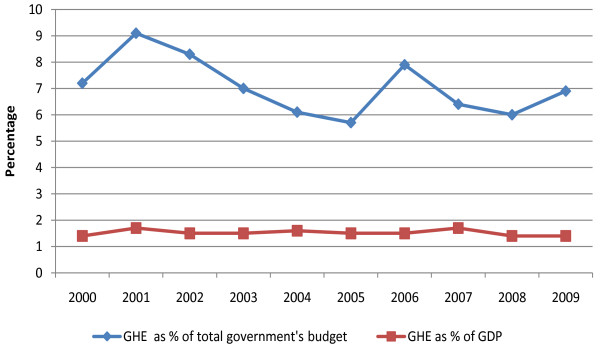
**Government health expenditure as proportion of government's budget and GDP**.

Figure 1 shows government's allocation to the health sector. In 2005/2006, government health expenditure (GHE) accounted for 5.73% of total government's budget. This proportion increased to 7.9% in 2006/2007. In 2007/2008, GHE as a percentage of government's budget declined to 6.4% and to 6.0% in 2008/2009. It was expected to increase to 6.9% in 2009/2010. Total government health expenditure as a share of GDP has remained below 2% in the last decade. There has been an increase in total government expenditure on health over time [18,22-26]. For example, between 2003/2004 and 2006/2007, total GHE increased from US$ 215.8 million to US$ 373.8 million. In 2007/08, total GHE declined by 21.1%, but increased by 54% in 2008/09. Estimates for 2010/11 and 2011/12 show an expected large increase in total expenditure amounting to US$ 614 million and US$ 673.9 million respectively [26]. Per capita expenditure increased from 5 US$ in 2000/01 to 13.8 in 2007/2008, declined to US$ 10.6 in 2007/2008 and increased to US$ 11 in 2008/09. This increase reflects growth in absolute amount of expenditures allocated to health.

#### Out-of-pocket payments

**Figure 2:**
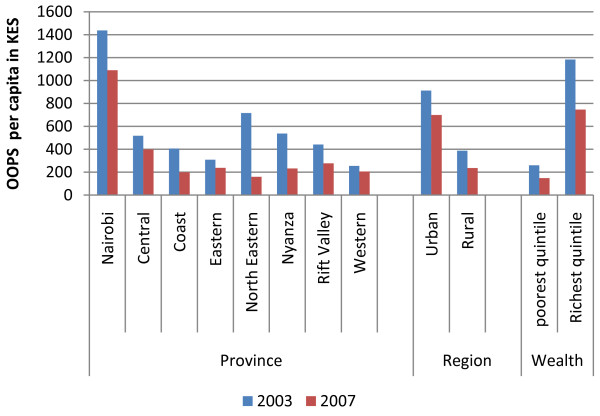
**Households per capita out-of-pocket spending (2003 and 2007)**.

The Kenyan health sector relies heavily on out-of-pocket payments (OOPs). OOPs are charged for health services sought from both the public and private sector. Out-of-pocket payments as percentage of total health expenditure accounted for 44.8% and 29.1% in 2001/2002 and 2005/2006 respectively. OOPs spending per capita amounted to Kenya shillings (KES) 819 (US$ 11.7) in 2003 and KES 578 (US$ 8.3) in 2007. Figure 2 shows the distribution of out-of-pocket spending for the years 2003 and 2007. Rural households reported significantly lower OOPs per capita of KES 387 (US$5.1) and 236 (US$ 3.1) in 2003 and 2007 respectively, compared to urban households who spent KES 912 (US$ 12) and 699 (US$ 9.2). Large differences in OOPs existed between provinces. The highest level of OOPs per capita spending was reported in Nairobi province in both 2003 and 2007 (KES 1436 and 1089 respectively), and the lowest reported in Western province (KES 255 and 205 respectively).

**Figure 3:**
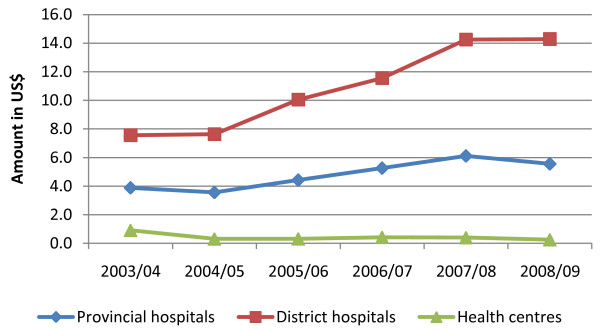
**Trends in user fees revenue**.

The amount of user fees revenue generated from public health facilities has increased gradually over the years. Total user fees collections amounted to US$ 13.2 million in 2003/2004. It increased to US$ 18.7 million in 2005. In 2008, total user fees revenue from all public health facilities amounted to US$ 25.7 million. Revenue collected at provincial and district hospitals show an increasing trend since 2003, while revenue collection at health centers and dispensaries has been on the decline (Figure 3).

#### Health insurance

Health insurance in Kenya is very low and comprises both mandatory and voluntary insurance schemes. Only about 10% of Kenyans have health insurance. Health insurance coverage is higher among the urban population (19.7%), compared to the rural population (7.4%); and among the richest (26.4%) compared to the poorest population (1.9%) [15].

The National Hospital Insurance Fund (NHIF) is the main type of health insurance in Kenya. It was established by an act of parliament in 1966 as a department under the ministry of health (MOH). The NHIF has undergone a series of restructuring over the years. For example, it was initially set to offer health insurance coverage to formal sector employees only, but in 1972, the Act was amended to incorporate voluntary membership, although in practice voluntary membership was only implemented in 2005. In 1990, the Act was repealed to allow contribution on a progressive basis and in 1998 the NHIF was transformed into a state corporation, delinking it from the MOH. The NHIF Act also provides for hospitals to get loans from NHIF to improve on service provision. Membership to the NHIF is mandatory to those working in the formal sector (both public and private) and voluntary for those working in the informal sector. Contribution to the NHIF ranges from KES 30 (US$ 0.4) for the lowest income groups, to KES 300 (US$ 3.8) for individuals earning above KES 15000 per month. Historically, these contribution rates were structured progressively (i.e. the rich contributed a higher proportion of their income than the poor), but this progressivity no longer exists since contribution rates have not been reviewed for the last 44 years of NHIF existence, while salary levels have increased significantly over time.

Voluntary health insurance- whether formal or informal- is very limited in Kenya. NHIF offers membership to the informal sector workers on a voluntary basis. Contributions for the informal sector are a flat rate of KES 160 per month (US$ 2.5). This suggests that informal sector NHIF contributions are regressive since the sector is very diverse and consists of both wealthy and poor populations. Following the introduction of user fees, a number of community based health insurance schemes (CBHIs) were initiated through donor support. Most of these CBHIs were not sustainable and discontinued operations following the withdrawal of donor funds. There are about 32 registered CBHIs in Kenya [27]. Community Based Health Insurance schemes in Kenya mainly operate in rural areas and are relatively small, undermining the potential for risk pooling and cross-subsidisation.

Private health insurance has been developing fairly well in recent years. There are three types of private health insurance providers in Kenya [28]: (1) General insurance companies that are involved in a wide range of insurance, not related to health, but who to a small extent insure people against ill health; (2) those that run medical schemes and are also health care providers operating their own clinics and hospitals where their clients seek care, although the same facilities are open to non-premium holders; (3) those that provide health care through third party facilities, also known as health management organisations, which are widely used for employer based insurance. In 2007, there were 14 private insurance companies offering health insurance in Kenya, with membership of about 600,000 people. Due to the high cost of premiums, membership to private health insurance comprises of the wealthiest population and is highest in the urban areas. For example, in 2007, 24.7% of Nairobi residents reported having private health insurance, while membership in other provinces was less than 5% [15]. Contributions to private health insurance are likely to be progressive, but although this can be cited as a positive element of this financing mechanism, it is only the members of these insurance schemes who benefit from it. Moreover, private health insurance often cream skim and fail to cover people with chronic conditions like HIV/AIDS, or when they do, the premiums are unaffordable. Consequently, people suffering from long-term illnesses cannot buy a cover-even when they can afford one- and they are therefore left to rely on public care which is already under-resourced. As a way of encouraging membership to private health insurance, individuals with private health insurance cover benefit from tax relief. This means that the poor tax payers subsidize health care for the rich, a factor that promotes inequities in the system.

### Collecting organisations

General taxes are collected by the Kenya Revenue Authority. There has been a steady increase in the amount of revenue collected since 2002, a factor associated with better revenue collection systems including electronic tax returns and strict penalties on defaulters. The local government also collects revenue that is used to support infrastructure at the local level. A proportion of this revenue funds approximately 100 health facilities, which are owned and operated by the local government. OOPs are collected by both private and public health care providers directly at the health facilities where care is sought. Government health facilities are allowed to retain 75% of revenue and the remaining 25 percent is forwarded to the district to support promotive and preventive services in the area. The NHIF collects monthly contributions from all members through payroll deductions for formal employees. Voluntary members make their personal payments through districts and provincial offices. Other collecting organisations include CBHIs and private health insurance schemes.

### Pooling of health care resources and allocation mechanisms

There is very minimal risk pooling in Kenya and hence, very limited cross-subsidisation. Apart from tax funding, other forms of pooling include the NHIF, private health insurance, CBHIs and to some extent donor funding where funds are channelled through the general budget support. Only 4% of all health funds are pooled through health insurance. The NHIF operates as a single risk pool making it the largest risk pool in the country. There are about 2 million workers and 8 million dependents covered by the NHIF, which amounts to about twenty per cent of the population [29].

Public funds are transferred from the Ministry of Finance to MOMS and MOPHS, who in turn transfer funds to the districts. Districts develop annual operations plans and prepare budgets for the year, which they submit to the district headquarters for consideration. The annual operation plans and budgets form the basis for resource allocation to the facilities. There exist two resource allocation formulas in Kenya, designed to allocate resources to primary level facilities (dispensaries and health centres) and district hospitals. The formulas include variables related to population structure, disease burden, infrastructure, poverty levels, utilisation patterns and hospital capacity. Informal discussions with MOMS and MOPHS officials suggested the formulas are hardly applied and were initially developed to allocate operation and maintenance costs only. Historical incremental approach remains the main basis of resource allocation in Kenya (Personal communication, Ministry of health official). Detailed information on the formulas are presented in Table 2.

**Table 2:** Resource allocation formulas for Kenya

Health centres and dispensaries	
**Variables**	**Weight**

Infrastructure	0.15

Under fives	0.2

Poverty levels	0.3

HIV/AIDS cases	0.05

Female population (15-49 years)	0.2

Area (square kilometre)	0.1

**Total**	**1**

**District hospitals**	

Poverty	0.2

Beds utilised	0.4

Out-patient cases	0.2

Accident prone facilities	0.05

Fuel costs	0.15

**Total**	**1**

Contributions to voluntary health insurance are allocated either through individual risk (for private health insurance) or community rated payments for CBHIs. Donor funds are mainly allocated directly to projects, although some donors fund directly through government budget support. Donor funds that are pooled through the government budget are allocated using the historical incremental approach. Although no figures were available on the amount of donor funds channelled through the government budget, informal interviews with MOH officials suggested that they are minimal since most donors are reluctant to fund through budget support. Discussions have been ongoing for a long period about the need for development partners to adopt a sector wide approach to funding the health sector, where all external funds are pooled and reallocated to priority areas, but progress has been slow (personal communication, MOMS and MOPHS officials). The criteria used by donors to target specific regions is not clear, but for disease specific projects, donor funding in Kenya has mainly focused on HIV/AIDS, malaria and to a small extent, reproductive health.

### Purchasing, provider payment mechanisms and benefit package

Health services in Kenya are purchased by different organisations through various mechanisms. The main purchasers are the MOMS and MOPHS, which operate 191 government hospitals, 465 health centres and 2122 dispensaries [18]. Other purchasing organisations include local government, NHIF, CBHIs, private health insurance and employers [19].

Public sector facilities are allocated budgets and staff are paid salaries using pooled tax funds. Some donors have allocated money to the Kenyan government to support the employment of nurses in remote rural areas. Donor funds under this arrangement are used to pay nurses salaries employed on a contract basis. There is no agency responsible for ensuring that public funds allocated to health providers are used appropriately. The NHIF works closely with public and private health facilities country wide to ensure all members have access to quality services. There are about 400 accredited health facilities that offer generalized, specialized and emergency healthcare services. Hospitals are accredited using a criteria that takes into account the range of services provided within a facility, personnel, bed capacity, infrastructure and equipment [30]. Payment to providers accredited to NHIF is through a flat daily rate. The reimbursement rates are determined based on the range of facilities available including X-rays, intensive care unit, number of health personnel, laboratories, operating theatres, overall area occupied, number of wards and ambulances [30]. Hospitals with large bed capacity and which offer a wide range of services receive higher reimbursement rates than smaller hospitals.

All Kenyans are entitled to health care provided through government facilities if they can pay user fees. Through tax revenue, the government subsidises all services provided in public health facilities and also meets the costs of waivers and exemptions for specific groups of the population (for example, children under five are exempted from any user fees, ante-natal services and specific heath conditions including tuberculosis, leprosy, psychiatric services do not attract any user fees). The Kenyan public health care system faces many challenges and while people are entitled to benefits provided through the public health care system, the reality is that good quality services are not always available [31]. Benefits arising from OOPs are proportional to the amount of money paid and are only accessible to the person making payment.

The NHIF act of 1998 entitles members to both outpatient and inpatient benefits although the former has not been implemented. NHIF provides an inpatient cover of up to KES 396,000 per year for the contributor, spouse and children. It covers up to 280 inpatient days per member and their beneficiaries each year [32,33]. The benefit package for the NHIF differs by type of facility, but it covers all diseases and maternity care. All government facilities, including teaching and referral hospitals, provide comprehensive cover to NHIF members without any copayments [33]. Individuals seeking care from faith based facilities and some small size private-for-profit facilities also enjoy comprehensive benefits, but a copayment of KES 15000 may be charged in cases of surgery, at the discretion of the health facility. Benefits at private facilities include a flat daily payment rate that differs depending on the size and kind of services available at the hospital and ranges from KES 400 to KES 1800 [32]. The reimbursement rates often form a small proportion of the total costs of care and people seeking care from private hospitals have to meet the remaining costs through OOPs or other forms of payment including private health insurance or employers related medical schemes.

Benefit packages under private health insurance are premium rated and vary from basic packages normally affordable to middle income groups, to sophisticated packages that are mainly designed to meet the needs of the richest populations. Benefit packages for CBHI members mainly involve inpatient care and are often linked to specific health providers, usually private-not-for profit or public health facilities. Private-for-profit services rarely provide services to CBHIs due to their high cost. Benefits related to donor funds are project specific and since it is not always clear how decisions are made in terms of what projects to fund and in which settings; it is difficult to exclusively assess the benefit packages under different projects. An overview of the Kenyan health financing system in terms of its key functions identified through the Kutzin [6] framework is presented on Appendix 1.

### Responding to equity challenges: Policy developments to address inequities in the Kenyan health system

The Kenyan government has a commitment to promote equity. A series of reforms are under way to address equity challenges in the Kenyan health system. Key among these reforms is the development of a health financing strategy [34] and the sector plan for health (2008-2012) [35]. Both documents highlight equity in access to health services as a priority area. The financing strategy aims at an equitable financing system, documents the policy options and highlights priority health sector reforms for achieving universal health coverage. Specific actions highlighted in the strategy include: implementing a national health insurance scheme; channelling funds directly to health facilities without passing through the district; increasing resources to underserved and disadvantaged areas and; scaling up the output based approach of financing (OBA) to include a range of health services (currently OBA in Kenya focuses on reproductive health services). The financing strategy is still in the early stages of implementation and it is not possible to assess the extent to which it will promote equity. Nonetheless, developing the financing strategy is an important step towards promoting equity and universal coverage in Kenya.

User fees at dispensaries and health centres were eliminated in 2004 and a flat registration fee of KES 10 and KES 20 respectively was introduced. Evidence suggest that user fees continue to be charged but charging levels remain lower than they were prior to the policy change [36,37]. Other reforms targeting the primary care level involve ensuring that these facilities receive their budget allocations on time. Health centres and dispensaries have in the past spent less than half of their budgetary allocations and cited delays in receiving funds from the district as one of the major reasons for the under spending [26]. For example, in 2008/09, dispensaries and health centres only spent 36.7% of budget allocations. To address this problem and also ensure that facilities are compensated for lost revenue arising from user fees removal, the government has introduced the health sector services fund (HSSF). Details on the HSSF are provided on Table 1.

Kenya is currently considering introducing a national health insurance, which will include transforming the NHIF to cover all Kenyans. The national health insurance proposal drew attention both locally and internationally in 2004/2005 but was met with resistance from various stakeholders including political leaders, employees and employers and private insurance companies. The Bill was highly controversial, it was nevertheless passed in parliament, but the president declined to sign it due to a mix of both technical and political reasons. Discussions on national health insurance were initiated again in 2007 and in September 2008, the Minister for Medical Services announced that the preparations for the scheme were at an advanced stage. Under the proposed scheme, the National Social Security Fund (NSSF) will be reformed and will purchase comprehensive health insurance for all contributors to the fund. Contributions will be income rated, with rates ranging from 2 to 2.5% of employees monthly salary. The bill has however not been taken to parliament.

The NHIF has been going through a series of reforms in recent years as part of its transformation process to a national health insurance scheme. The introduction of a national health insurance scheme is regarded as the catalyst for achieving universal coverage in Kenya. Recent changes include: (1) actively seeking to increase coverage among the informal sector through conducting outreach activities in both urban and rural areas. (2) The scheme provides a comprehensive cover to those seeking inpatient care in government and faith based facilities; (3) Plans are under way to broaden the benefit package to include outpatient care. A pilot project was conducted in a rural and urban district to assess the impacts of an outpatient cover on utilisation patterns and costs. (4) A revision of the contribution rates has been proposed. Current contribution rates were set in 1966, and have not been revised to take into account the high costs of services and inflation. The new rates are structured progressively and involve an increment of over 500 per cent for high income earners [38]. These rates were required to be implemented in September 2010, but they have faced strong opposition from trade unions, and the matter is currently in the high court [39].

Other recent NHIF reforms include decentralisation to ensure that services are close to the people. Until recently, the NHIF was highly centralised in Nairobi where all claims were processed. Health facilities in the rest of the country were required to make monthly trips to Nairobi to pursue their claims, leading to high transaction costs for members and health care providers. The NHIF has now decentralised claims processing to district offices to facilitate a shorter and more effective system that will allow speedy reimbursement of medical claims. The NHIF has so far opened 28 branches across all provinces, in both rural and urban areas. Other changes include simplified, computerised claim procedures and the establishment of an electronic data base. These improvements have made it easier for members and health providers to make claims faster and at a relatively low cost.

## Discussion

This paper set to examine the Kenyan health system in terms of key financing functions namely: revenue collection, pooling and purchasing. Here, the main findings are discussed and recommendations made on how the Kenyan health system can make progress towards universal coverage.

The results show that out-of-pocket payments remain the main sources of health care funds in Kenya. The negative consequences of OOPs are well documented [11,13,17,40-44]: they are regressive; are considered the worst form of health care financing; they lead to catastrophic financial payment and impoverishment, especially among the poor; and are a major barrier to health care. Waiving mechanisms introduced to protect the poor from paying user fees have not been effective in Kenya or other parts of Africa [10,11,45]. Efforts have been made to reduce user fees at the primary care level where varying charges were replaced with a standard fee of KES 10 and KES 20 for dispensaries and health centres respectively. While this is an important step towards promoting access to health care among the poorest populations, similar reforms should be implemented at the hospital level. Strong commitments to move away from user fees and other forms of OOPs towards tax funding and health insurance are required. However, it is worth noting that the majority of Kenyans work in the informal sector and poverty levels are high; suggesting that even with the introduction of mandatory health insurance, a large number of Kenyans would still require to be fully covered through tax funding. Caution should therefore be taken when introducing any financing reforms to ensure that the needs of the poor and vulnerable are protected and that they are implemented within the context of universal coverage [46].

An important indicator of government's commitment to health is the proportion of government's budget allocated to the sector. In 2001, African heads of states met in Abuja and committed to allocating at least 15% of annual budgets to the health sector. Government spending on health in Kenya is less than half the Abuja target [26] and has been declining, in addition to being the lowest in East and Southern Africa [47]. Although very few African countries have achieved the Abuja target, most countries are slowly increasing their allocation to the health sector [47], with the exception of Kenya. The WHO commission on Macroeconomics for health made a case for more investment in health to attain the average of US$ 34 per capita expenditure needed to make health care accessible to the entire population [48]. There has been a steady increase in Kenya's government health expenditure per capita from US$ 5 in 2000/2001, to US$ 13.4 in 2007/2008. This increment reflects the growth in absolute terms of government's allocation to the health sector. When donor funds are incorporated into the analysis, total health expenditure per capita increased significantly to US$ 27 in 2005/2006 [19]. Although the commission recommended that developing countries be supported by donors to achieve the US$ 34 target, the increase in per capita spending on health in Kenya is largely due to an influx in donor support for HIV/AIDS. Per capita spending would be significantly lower if HIV/AIDS related funds were excluded from the analysis. While donor funds have significantly contributed to better access to health care in Kenya, particularly for people living with HIV/AIDS, they should supplement but not replace government funding.

The Kenyan government should aim at gradually increasing their share to the health sector to avoid serious drawbacks should donor funds be significantly reduced or suspended. Various alternatives can be adopted to increase the proportion of government funds allocated to the health sector. First, the government can simply increase the share of budget allocated to health, and in so doing reduce the percentage share allocated to other sectors. While this may appear relatively straightforward, it can have some challenges including, opposition from other sectors that are also advocating for more resources. Health is influenced by many other factors beyond the ministries of health for example, education, agriculture, and housing. Reducing the share allocated to these sectors could negatively impact on the health status of the population. Nevertheless, some negotiations should be done to increase the share of health spending in a way that does not undermine other sectors influencing health. Secondly, the government can direct efforts towards increasing the amount of revenue collected through strengthening taxation of corporate and personal incomes, and allocate a large proportion of the increased revenue to the health sector. Interestingly, the Kenyan government has been recording massive increments in the amount of tax revenue, but this has not translated to any noticeable increment to the percentage share of government budget allocated to health. Thirdly, earmarking some taxes for health care might ensure that a certain proportion of government revenue is protected for the health sector, and in so doing increase the budgetary allocations to the level required to provide the essential package for health.

The Kenyan health financing system is very fragmented. Fragmentation refers to the existence of a large number of separate financing mechanisms and a wide range of health care providers in a country [49]. It exists when funds collected from different financing mechanisms are not pooled and people from different socioeconomic status are covered under different arrangements [49]. OOPs present the main form of fragmentation in the Kenyan health system. Other forms of fragmentation exist in the form of NHIF, CBHIs, private insurance and donor funding. The NHIF mainly covers people working in the formal sector; private health insurance companies cover the high income groups, while most CBHI members are small scale farmers. There is very limited income cross-subsidisation in CBHIs and private health insurance since members are of similar socio-economic status, and they often exclude the poorest. For example, each of the 32 CBHIs in Kenya functions independently, resulting to very small pools that offer limited protection to a minority of the population. Plans are underway to integrate all CBHI schemes in Kenya, but it is still unclear what aspects will be managed under the larger pool. Although the NHIF enjoys high membership, these funds are not pooled together with CBHI contributions, or with tax funding. The local government also collects revenue and funds 100 health facilities country wide. Revenue from the local government is not pooled with tax funds allocated to the ministries of health. Donor funds are also very fragmented; most projects operate independently, and it is common to have different donors funding similar health projects within the same district, but with little, if any, cooperation in terms of financing, operations and service delivery. Funding specific health programmes independently undermines sustainability of health financing. The WHO has called for better coordination of donor funds to ensure that external funds are consistent with countries priorities and within the broader objective of universal coverage [4]. Failure to pool donor resources in Kenya promotes inequities because they are not considered when government allocations are being made, especially where regions with less need benefit from significant donor funding and also receive a large share of government funding.

Fragmentation is not unique to Kenya. Health systems in low and middle income countries have in the past been highly fragmented. Some countries including Ghana, Kyrgyzstan, Thailand, are making progress towards universal coverage under a less fragmented system. Other countries including South Africa and Tanzania are in the process of implementing major reforms that will promote harmonization and universal coverage [49-51]. Various authors highlight the need to harmonize health care financing arrangements if universal coverage is to be achieved [46,49,51]. They note that health financing should be approached from a systemic perspective that is informed by policy goals, rather than implementing piece meal reforms, which focus on each source of funding independently and in so doing promote fragmentation and segmentation. It is clear that past and current health reforms in Kenya have not adopted a systemic approach to health care financing. A potential starting point for promoting a unitary system is to pool NHIF funds with tax funding and purchase health services centrally. Linking CBHIs and other microfinance institutions offering financial risk protection to those outside the formal sector with the NHIF, in order to maximise income cross-subsidisation would also promote harmonisation. Ensuring that donor funds are integrated in the health system to ensure a coherent approach to health care delivery is also critical.

The way health care resources (tax, donor funds, health insurance) are allocated to purchase health care services has implications for equity and universal coverage. Allocation of public health funding in Kenya is done on a historical incremental basis. Resource allocation formulas exist, but they are hardly applied. Relying on an incremental approach to allocate funds promotes inequities since demand and supply factors are the major determinants of budgetary allocation. Historically advantaged regions (i.e. those with high number of health facilities), receive larger budgetary allocations than disadvantaged regions with less health facilities. The Kenyan government should ensure that resources are allocated according to need. A potential starting point is to review the formulas presented in Table 2. The main indicators captured in the current formulas relate to infrastructure and utilisation patterns, which suggest that they allow for historical allocation. Need based indicators that have been widely shown to promote equity in resource allocation including population size, infant mortality and under five mortality are not included in the formulas [49]. An important element of ensuring that equity is achieved and that need based resource allocation is widely accepted is to estimate equity targets for each hospital or geographical region. These equity targets should guide reallocation of resources in a phased manner to ensure that facilities make adjustments in preparation for budgetary increments or reductions and that opposition is minimised.

Donor funds constitutes a large proportion of health expenditure and depending on how they are allocated, they can promote or hinder equity. Where funds are channelled directly through project funding, inequities can exist, especially when donors show preference for one geographical location based on purely practical and historical reasons rather than need for care. Channelling donor funds through the budget support and ensuring that the same are allocated to different regions using a need based formula can promote equity. This however requires that the government can be trusted by donors to spend funds efficiently. Nevertheless it is important that donor funds are allocated and managed in a way that is consistent with the broad objective of the Kenyan financing system in order to support the country's move towards universal coverage.

Only a minority of Kenyans have insurance cover. The majority of Kenyans with health insurance cover work in the formal sector and comprise the richest population. The NHIF is the main source of insurance cover for individuals working in the formal sector, and although it allows voluntary membership for informal sector workers, coverage levels remain low [15]. Plans are under way to introduce a national health insurance scheme that offers financial protection to all Kenyans. While this is a good development, it remains unclear when this policy change will be implemented. More important, how to provide coverage to those working in the informal sector remains a major challenge for universal coverage in Kenya. The challenges of increasing health insurance coverage among informal sector workers are well documented. Thailand, for example, made slow progress towards universal coverage for many years, until the government decided to purchase premiums for informal sector insurance using tax funds [52]. As a part of the preparation towards implementing the new financing strategy for universal coverage, the Kenyan government should reconsider the equity implications of covering informal sector workers through contributory health insurance versus tax funding. Such assessments should form the basis of moving the financial debates in the country forward.

Until the translation to a national health insurance fund is implemented, the NHIF will remain the main source of health insurance in Kenya. The NHIF plays an important role in protecting households from high inpatient related costs, but the poor and those working in the informal sector do not benefit from its services. Efforts to increase NHIF coverage among those working outside the formal sector have achieved limited success. Consequently, there is very limited income cross-subsidisation in NHIF. The proposed national health insurance scheme is regarded as the main mechanism towards universal coverage. However, it is not clear how the 'new' national insurance will address the low coverage levels experienced by the NHIF.

The government together with NHIF should identify ways of how to best fund premiums for informal sector workers, either through government tax or donor support. Should donors commit to contributing towards premiums for the informal sector, this should be a short term strategy as government puts measures in place to offer financial protection to all its citizen.

The NHIF offers a comprehensive benefit package to members seeking treatment in government and faith based hospitals. Those seeking care from private hospitals often incur OOPs since the benefit package only meets a small percentage of inpatient costs. Large co-payments undermine the financial risk protection provided through health insurance. It is important to design an affordable and sustainable benefit package with minimal or no copayments. Currently, the NHIF operates at a surplus and spends a large proportion of revenue on administration, while providing a very minimum benefit package. Restructuring the NHIF to minimise the administration costs can release funds for purchasing comprehensive services to members. Finally, any improvements in the NHIF benefit package should be done in consideration of the wider health system, to ensure that the goal of achieving universal coverage is achieved.

A major limitation to most of the past and present policy developments in Kenya is the failure to involve the public in the identification and implementation of policy interventions. Policies may have good intentions, but translating them into practice and ensuring that the intended gains are achieved can be a challenge [53]. The government should engage with the public when designing policies to promote universal coverage in order to ensure that their preferences are adequately considered. Engaging the public in early stages of policy design can promote acceptability and thus contribute towards effective implementation.

## Conclusion

The Kenyan health system is highly inequitable and policies aimed at promoting equity and addressing the needs of the poor and vulnerable have not been successful. There is less commitment to increase the proportion of government funds allocated to the health sector. Some progress has been made towards addressing equity challenge, including reducing user fees at primary health care facilities and developing a health financing strategy, but universal coverage is unlikely to be achieved unless the country adopts a systemic approach to health financing reforms. Such an approach should be informed by the wider health system goals of equity and efficiency.

## Competing interests

The authors declare that they have no competing interests.

## Authors' contributions

JC was responsible for the overall design of the study. JC and VO were involved in reviewing the literature and policy documents. Both authors read and approved the manuscript.

## Appendix 1: An overview of the Kenyan health financing system

### Revenue collection

#### Sources of funds

1. OOPs

2. General tax revenue

3. Social health insurance

4. Private health insurance

5. Donor funds to the government and private not-for profit organizations

6. Community based health insurance

#### Contribution mechanisms

1. Direct taxes: payroll tax deductions, structured progressively.

2. Indirect taxes

a. VAT charged at 16%, but some items are exempted from tax.

b. Corporate income tax ranges from 20% to 37.5%.

c. Custom duty tax ranges from 0% for exempted items to 40%.

d. Excise duties range from 0% to 120%

3. Out-of-pocket payment mechanisms

4. NHIF contributions

a. Payroll deductions for formal sector, payment at designated centres for members outside the formal sector.

b. For formal sector employees, contributions range from 30 to 320 Kenya shillings per month.

c. Informal sector members contribute 160 Kenya shillings per month.

5. Private health insurance

a. Contributions are risk rated.

6. Community health insurance

a. Community rated contributions that are decided following consultations with community members.

#### Collecting organisation

1. All taxes and payroll contributions/deductions are collected by the Kenya Revenue Authority.

2. Mandatory insurance contributions are collected directly by the NHIF.

3. Private health insurance companies are paid directly by the employer or individual clients to insurance companies.

4. CBHIs have localized collection mechanisms.

### Risk pooling

#### Coverage and composition of risk pools

1. NHIF covers all individuals working in the formal sector and their dependents aged below 21 years. Children aged above 21 years and who are still in school are also entitled to benefits from their parents' membership. People in the non-formal sector can join on a voluntary basis. There are approximately two million members from the formal sector, and 250000 from the informal sector.

2. There are no risk pools with OOP payments and only those who can afford receive health services

3. Private health insurance mainly covers the wealthier population. Recently, private health insurance companies have developed products that are affordable to middle income groups, but uptake remains low.

4. CBHIs membership is limited and composed of small scale farmers in rural areas.

#### Allocation mechanisms

1. Public revenue is allocated using a historical approach. Two resource allocation formulas exist.

2. Expenditure patterns account for disease burden but emphasis is currently shifting to the human capital approach to health.

### Purchasing services

#### Benefit package

1. *Service type*

a. NHIF covers inpatient cover for all illnesses including emergencies. It offers a comprehensive benefit package to members seeking care at public hospitals. Those seeking care at faith based facilities also enjoy comprehensive services but are required to make a copayment of KES 15000 in case of surgery. For private-for-profit facilities, individuals are entitled to a daily rate payment, which ranges from KES 400-2000 depending on the accreditation agreement.

b. Private insurance schemes provide cover according to an individual's premium based on ability to pays. The packages are predetermined and potential clients choose a package whose costs they can meet.

c. CBHIs mainly offer inpatient cover for all illnesses at specific health facilities.

2. *Type of provider*

a. The NHIF accredits both government and private health facilities.

b. Insurance companies either contract hospitals, run own facilities (HMOs) or pay reimbursement to providers for services rendered to clients.

c. CBHIs mainly work with faith based hospitals within their catchment areas although a few offer coverage in government hospitals.

3. *Affordability and sustainability*

*a. *NHIF packages are standard for all beneficiaries and are affordable to those working in the formal sector. Copayments remain a main barrier to access and promote inequities.

b. Most high income earners can afford comprehensive individual insurance from private companies

#### Provider payment mechanisms

1. Public providers are allocated a budget and employees paid monthly salaries

2. Various private health insurance companies pay on a case-based fees or fee-for-service to accredited hospitals; others also provide services and pay them employees salaries

3. Faith based facilities are allocated budgets by donors and often charge a flat fee for basic illnesses

4. For OOPS, households pay fee-for-service to individual providers

### Service provision

1. The government is the main health care provider, but there exists a significant private sector.

2. The NHIF purchases services from both public and private health providers

3. There is a large number of independent providers without contracts
